# Lipedematous alopecia: clinical and histological analysis of the first male Chinese

**DOI:** 10.1186/s40064-016-3430-8

**Published:** 2016-10-10

**Authors:** Wei Wang, Guizhi Zhou, Yanfang Zhang, Changping Yu, Furen Zhang

**Affiliations:** 1 Shandong Provincial Hospital for Skin Diseases, Shandong University, Jinan, 250022 Shandong Province People’s Republic of China; 2 Department of Dermatology, Jining No. 1 People’s Hospital, Jining, Shandong Province People’s Republic of China; 3 Shandong Provincial Institute of Dermatology and Venereology, Shandong Academy of Medical Sciences, No. 27397 Jingshi Road, Jinan, 250022 Shandong Province People’s Republic of China

**Keywords:** Lipedematous alopecia, Male, Histological examination, Skin biopsy

## Abstract

**Background:**

Lipedematous alopecia (LA) is a rare disorder clinically characterized by a thick boggy scalp and varying severity of hair loss, which primarily occurs in black female adults. In this study, we reported one male adult Chinese diagnosed with lipedematous alopecia for the first time.

**Case presentation:**

A Chinese male, aged 20 years old, admitted to Shandong Provincial Hospital for Skin Diseases was diagnosed with LA. Clinical, histological and imaging data were collected and analyzed. Literature review was performed.

**Results:**

Skin biopsy and pathological examination revealed the signs of increased subcutaneous adipose tissue, slight perivascular mononuclear infiltration into superficial dermis, adipocyte disruption and mucin deposition. CT scan demonstrated diffuse thickening of subcutaneous tissue in the occipital region. The symptoms were not significantly alleviated after 6-month combined therapy of prednisone and acupuncture.

**Conclusions:**

We reported one male Chinese of LA, who was the first case reported in China. Clinical and pathological findings of this case deepened the understanding of LA.

## Background

Lipedematous scalp is a rare condition manifested as an increased thickness of subcutaneous tissue in the scalp gives rise to a soft spongy appearance of the surface and occasionally causes pruritus and pain in the affected area. When hair loss is also associated with the condition, it is described as lipedematous alopecia (LA). LA is a rare condition characterized by a thick, boggy scalp with varying degrees of hair loss that occurs in adult black females, with no clearly associated medical or physiological conditions. However, the underlying pathogenesis remains elusive (Martin et al. 2005; Ko et al. 2011; Fair et al. 2000). Pathologic finding reveals an approximately doubled scalp thickness resulting from expansion of the subcutaneous fat layer in the absence of adipose tissue hypertrophy or hyperplasia. Interestingly, LA is primarily encountered in the female. In this study, we firstly reported one male Chinese patient diagnosed with LA. Clinical, pathological and imaging features were retrospectively analyzed, adding clinical evidence to understand of this rare clinical entity.

## Case presentation

**Fig. 1:**
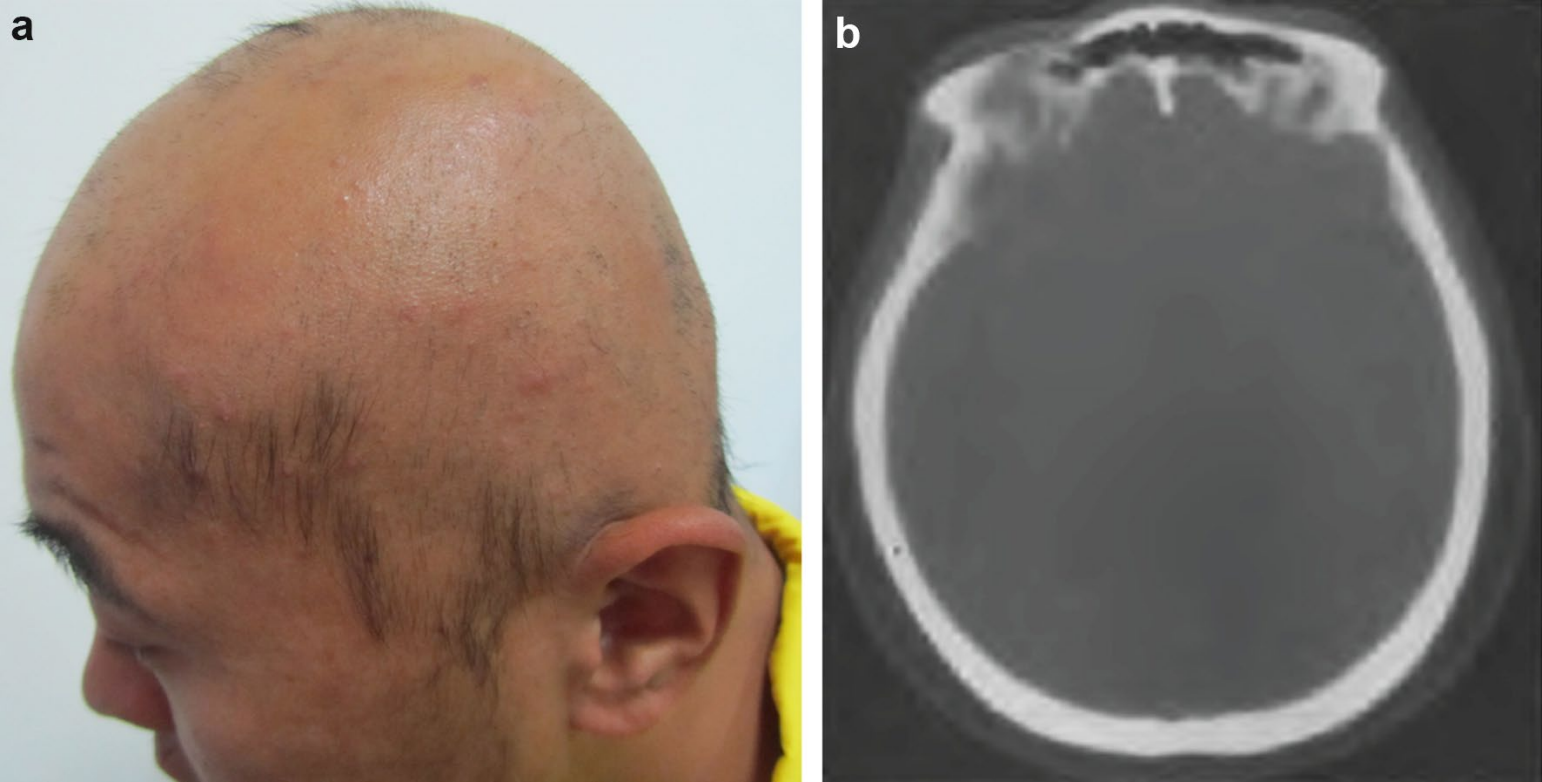
**a** Clinical manifestation showing alopecia accompanied by thickened scalp. **b** CT scan showing thickening scalp with marked expansion of subcutaneous adipose tissue approximately 15 mm in the occipital region

A 20-year-old Chinese man presented with a 4-year history of scalp pruritus and hair loss. At the age of 16, he started to suffer from a gradual loss of hair, initially from the vertex and occipital areas, but eventually extending to the entire scalp during subsequent 1 month after the onset of symptoms. He reported no family history of a similar entity or trauma. Physical examination revealed alopecia of the entire scalp and the remaining hair was short and less than 2 cm, as shown in Fig. 1a. Upon palpation, the scalp felt mildly tender and had a boggy spongy consistency. The scalp could be easily pressed down to the underneath bone, but immediately restored to original shape. Computer tomography (CT) scan demonstrated diffuse thickening of the subcutaneous tissues in the occipital region, which was measured as approximately 15 mm (Fig. 1b).

**Fig. 2:**
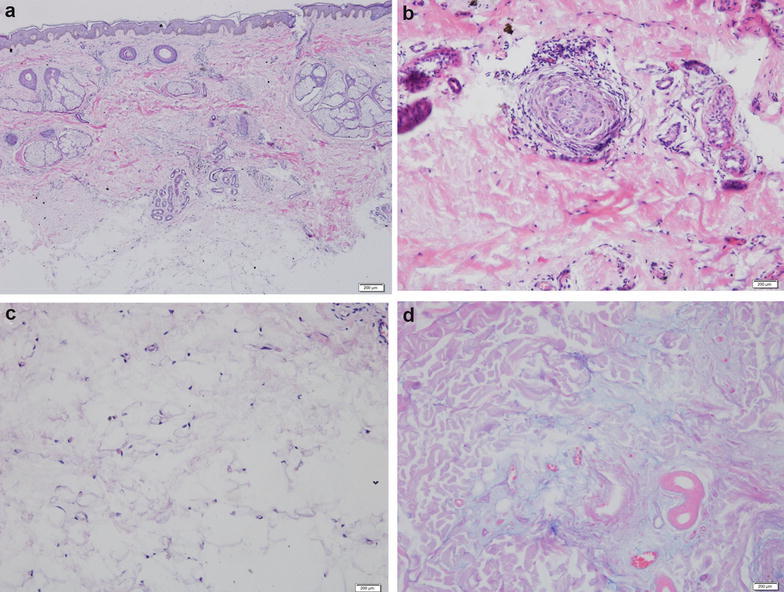
**a** H&E staining revealing mild perivascular mononuclear infiltration into the superficial dermis and increased subcutaneous adipose tissue (×100 magnification). **b** H&E staining showing perifollicular fibrosis (×400 magnification). **c** H&E staining revealing disruption of adipocytes (×400 magnification). **d** Alcian blue staining showing mucin deposition in the dermis (×400 magnification)

Chemical panel, complete blood count, thyroid function test, and antinuclear antibody titers yielded normal outcomes. Trichoscopy revealed no yellow dots, black dots, or exclamation mark hair. Hair pull test was negative. No hair loss was observed in other body parts. Skin biopsy showed increased subcutaneous adipose tissue and mild perivascular mononuclear infiltration into the superficial dermis (Fig. 2a). Scant perifollicular mononuclear infiltration accompanied by perifollicular fibrosis was equally observed in Fig. 2b. The count of hair follicle was decreased. Adipocyte disruption and mucin deposition were also demonstrated, as illustrated in Fig. 2c, d.

According to the findings and outcomes, he was eventually diagnosed with LA. After the diagnosis was validated, the patient was administered with prednisone at a daily dosage of 30 mg in combination with acupuncture therapy for consecutive 6 months. Acupuncture therapy has been reported to accelerate the blood circulation and stimulate the acupoint. However, the symptoms of hair loss and the thickening of subcutaneous fat tissue were not significantly mitigated after this combined therapy.

## Discussion

LA is an extremely rare condition firstly described by Coskey in 1961, dominantly occurring in black female adults (Kavak et al. 2008; Blaheta et al. 1999). To date, more than 80 LA cases have been reported in the literatures (Müller et al. 2012), a majority of whom are black female patients including merely three Asians. Moreover, LA is primarily encountered in female patients, and only four male cases have been reported yet. Common symptoms of LA include joint and skin hyperelasticity, discoid lupus, diabetes mellitus and acute kidney failure (Ko et al. 2011). LA is pathologically characterized by increased subcutaneous adipose tissues (Yasar et al. 2011). The scalp thickness of LA varies from 10 to 16 mm, significantly thicker compared with 5.5 mm of healthy counterparts (Yasar et al. 2007).

In present investigation, we reported the first male patient diagnosed with LA in China, suggesting that the incidence of LA might be more geographically and ethnically widespread than previously thought (El Darouti et al. 2007). Moreover, the incidence of LA probably does not significantly differ between both genders. Histopathological findings revealed adipocyte disruption besides increased subcutaneous adipose tissue in this male patient, which has been seldom reported. We assumed that the incidence of LA may be related to adipocyte disruption, which warrants further investigation. Scant perifollicular mononuclear infiltration and perifollicular fibrosis are inconsistent pathological features of LA patients. To date, only two cases have been reported to have mucin deposition in the dermis or subcutis. The patient in present study presented with mucin in the dermis and subcutis. The causes and clinical significant of this sign remain to be further elucidated.

In our case, multiple symptoms including hair loss, thick and boggy scalp were found simultaneously and the entire scalp rapidly evolved with only 1 month, indicating that LS and LA are separate entities. Mounting evidence supported an autoimmune origin of LA, although the exact cause and triggering factors are still unknown. To validate the diagnosis of LA, clinical, histopathological and other findings should be performed simultaneously. Currently, multiple surgical (Yip et al. 2008) and medication therapies have been applied to alleviate the symptoms of LA and LS, whereas poor clinical efficacy has been achieved. Yip et al. successfully treat a 67-year-old woman with LA by using surgical debulking combined with scalp reduction and acceptable efficacy was obtained after 12-month follow-up (Cabrera et al. 2015). In a recent investigation, Cabrera et al. (2015) reported that a female patient diagnosed with LA was properly healed after administration of mycophenolate mofetil. Optical treatment of LA remains to be explored by large sampling size investigations.

## Abbreviations

LA: lipedematous alopecia
CT: computer tomography
